# Assessment of potential drug-drug interactions in hospitalized patients with infectious diseases: an experience from a secondary care hospital

**DOI:** 10.12688/f1000research.143186.4

**Published:** 2025-01-02

**Authors:** Javedh Shareef, Sathvik Belagodu Sridhar, Abu Nawa Ahmad Ismail, Padma G.M. Rao, Rashid Ain Ur

**Affiliations:** 1 Department of Clinical Pharmacy & Pharmacology, RAK College of Pharmacy, RAK Medical & Health Sciences University, Ras Al Khaimah, 11172, United Arab Emirates; 2 Internal Medicine Specialist, Ibrahim Bin Hamad Obaidullah Hospital, Ras Al-Khaimah, Ras al Khaimah, 11172, United Arab Emirates

**Keywords:** Drug interactions, polypharmacy, prescription drugs, prevalence, patients

## Abstract

**Background:**

Polypharmacy is common among hospitalized patients with infectious infections owing to comorbidities or concurrent illnesses. This raises the likelihood of drug-drug interactions and creates uncertainty for healthcare providers. This study aimed to assess the potential drug-drug interactions (pDDIs) among hospitalized patients with infectious diseases in a secondary care hospital.

**Methods:**

A prospective observational study was conducted in the internal medicine ward for six months. Data were collected from patient case records, and prescriptions were screened for pDDIs and classified based on the severity from a portable electronic physician information database (PEPID) resource analyzed using SPSS, version 27.0.

**Results:**

In total, 148 patient case records were analyzed, and 549 pDDIs were identified, with 66.8% having at least one or more DDIs. The mean number of drug interactions was 3.70 ± 4.58 per prescription. The most frequently encountered drug interactions were drug combinations such as bisoprolol with atorvastatin and aspirin with tazobactam/piperacillin. Based on the severity, most pDDIs belong to the ‘moderate’ category (40.07%). Bivariate analysis showed that age, comorbidities, length of hospital stay, and the number of drugs prescribed were risk factors associated with DDIs (p<0.05). In the multiple binary logistic regression analysis, DDIs were significantly associated with comorbidities and the number of prescribed medications (p<0.0001).

**Conclusions:**

This study observed the prevalence of DDIs in hospitalized patients with infectious diseases of ‘moderate’ severity. Prescription screening using a drug information database assists in identifying and preventing DDIs early, enhancing drug safety and quality of patient-centered care.

## Introduction

Infectious diseases are among the most common health concerns globally, regardless of age. Infected people frequently require hospitalization, which increases the risk of morbidity and mortality and raises healthcare costs. ^1^ Infectious diseases are conditions caused by microscopic organisms such as bacteria, viruses, fungi, or parasites that spread from one person to another. ^2^ Healthcare providers frequently face challenges in selecting and using antimicrobial medicines. ^3^^,^^4^

Drug-drug interactions (DDIs) occur when two or more co-administered drugs interact, with one drug altering the effect of the co-administered drug. The outcome effect of drug interactions may vary from non-serious to serious/life-threatening (or) irreversible, affecting the goals of therapy, clinical effectiveness, and worsening treatment outcomes. ^5^ Studies have reported that age (≥ 65 years), polypharmacy, increased number of prescribers, and comorbid illness are defined risk factors for drug interactions. ^6^^,^^7^ In addition, a decline in drug metabolism associated with aging, comorbidities such as hepatic and renal injury, and altered drug plasma concentrations complicate medication use and increase the sensitivity to drug interactions. As a result, clinically significant drug interactions prolong hospital stays, increase re-visit and healthcare expenditures, and aggravate patient outcomes in inpatient and outpatient healthcare settings. ^8^^–^^10^

Drug interactions are classified as pharmacokinetic and pharmacodynamic interactions; a few are categorized as unknown or other based on their mechanism of interactions. Drug interactions can be grouped as major, moderate, or minor according to severity and significance. ^11^ Studies carried out in different health settings and patients reported that potential drug-drug interactions (pDDIs) range from 19.3% to 91.6%. ^12^^,^^13^ A systematic review and meta-analysis reported that the prevalence of clinically manifested DDIs ranged from 1.2% to 64.0%. ^14^ The increased incidence of adverse outcomes associated with drug-drug interactions is a common cause of hospital admission, primarily in the aging population. ^15^ However, the variation in the results across different studies is associated with factors such as patient characteristics, prescribing pattern, severity of the illness, study population, and study setting.

The use of clinical decision support systems, close monitoring of patient’s drug therapy, and involvement of clinical pharmacists in a multidisciplinary team are some of the important measures that help to minimize drug interactions and improve patient safety. ^16^^,^^17^

Studies on antimicrobial agents in the United Arab Emirates (UAE) have focused on the prescription pattern of drug use and related outcomes in various hospital settings. However, studies related to DDIs with antimicrobial agents in infectious diseases are unaddressed despite being one of the reasons for hospitalization. Therefore, the present study was carried out to assess pDDIs among hospitalized patients with infectious diseases in a secondary care hospital.

## Methods

### Study design and study setting

This prospective observational study was conducted from March 2021 to August 2021 in the internal medicine department of Ibrahim Bin Hamad Obaidullah Hospital in the northern Emirate of the United Arab Emirates.

### Ethical approval

This study was performed per the principles outlined in the Declaration of Helsinki, the US Federal Policy for the Protection of Human Subjects (Common Rule), and the European Medicines Agency Guidelines for Good Clinical Practice. ^18^ Approval was obtained from the human ethics committee of Ras Al Khaimah Medical and Health Sciences University (RAKMHSU-REC-068-2020/21-UG-P) and the Research Ethics Committee of Ministry of Health & Prevention, Ras Al Khaimah (MOHAP/REC/2021/1-2021-PG-P) in January 2021.

After getting approval from the MOHAP-RAK REC, the principal investigator obtained written informed consent from all the patients who met the study criteria after explaining the study procedures and other details to the participants.

### Inclusion criteria

Hospitalized patients aged 18 years and older who were diagnosed with infectious diseases caused by bacterial pathogens and received a minimum of two or more medications containing at least one antimicrobial agent were included in the study.

### Exclusion criteria

Patients referred from other departments admitted to the intensive care unit, diagnosed with COVID-19 receiving antibiotics, with incomplete medical records, and pregnant or lactating were excluded from the study.

### Sample size and sampling technique

The sample size was calculated using the formula to estimate a single proportion [n = (Z – α/2) ^2^ p (1 − p)/d ^2^] where Z = standard normal variable at 95% confidence level (1.96), p = the prevalence of pDDIs assumed to be 50% and finally adjusted using a correction formula. The minimum sample size was 150 patients with 5-10% dropouts. Patients admitted during the study period were considered for the sampling frame and included using the systematic random sampling technique.

### Data collection

The medical records of the hospitalized patients who met the study criteria were reviewed daily. The data were collected from the Wareed system, an electronic health record information system (HIS), a technological platform that virtually connects all the government hospitals of ministry healthcare facilities in Dubai and the Northern Emirates by automating all healthcare processes across various departments. All necessary details of the patients, including drug therapy, were collected from the electronic health records and documented in the data collection form designed according to the needs of the study.

### Assessment of drug-drug interactions

All prescription medicines were added to the ‘drugs to check’ list in the portable electronic physician information database (PEPID) interaction tool for evaluating pDDIs. (Pepid. LLC, 2024) The identified drug interactions were classified by level of concern as minor/non-significant, minor, moderate, significant, and life-threatening. They were also based on pharmacokinetics, pharmacodynamics, and other/unknown mechanisms. ^19^ The severity of interactions in PEPID is represented by colored warning triangles stacked in descending order. The number value within each triangle relates to the severity of the interaction, with a value of “5” indicating a potentially fatal circumstance, and the combination should never be employed. Level 4 implies a major interaction, which has a high risk of being severe or lethal. Contraindicated unless the benefits outweigh the hazards and no other choices exist. Level 3 indicates a moderate interaction, necessitating strict monitoring and the use of alternate drugs, if possible. Furthermore, level 2 implies a strong contact that requires close monitoring, whereas level 1 indicates a minimal or insignificant interaction.

### Mechanism of interaction

Pharmacokinetic interactions can influence how medications are absorbed, transported, metabolized, and eliminated.

The term “pharmacokinetic drug interactions” describes modifications to a drug’s distribution, metabolism, excretion, or absorption brought on by the presence of another medicine. Due to these interactions, drug concentrations in the body may change, raising toxicity or reducing therapeutic effectiveness. Typical processes include competition for protein binding, changes in the pH of the gastrointestinal tract, and inhibition or activation of enzymes.

Pharmacodynamic interactions occur when the effects of one drug are altered by the presence of another at its site of action, potentially resulting in synergistic or antagonistic therapeutic function or undesirable side effects. These interactions may result in unforeseen side effects, diminished (belligerent), or boosted (synergistic) effects. They can affect the drugs' overall therapeutic success and safety profile. They can result from comparable modes of action, opposing effects, or interactions at the same receptor sites. ^19^

### Data analysis

The class of medications involved in the onset of pDDIs was analyzed using the Anatomical Therapeutic Chemical (ATC) classification system derived by the World Health Organization. ^20^ The collected data were scrutinized and checked for completeness, clarity, and legibility before being entered into a Microsoft Excel (RRID: SCR_016137) spreadsheet and were later analyzed using IBM SPSS Statistics (RRID: SCR_016479) version 27 (IBM Corp., Armonk, NY, USA). Descriptive statistics, such as mean, standard deviation, frequencies, and percentages, were used to describe continuous data. Bivariate analysis using a chi-square test was used to identify factors associated with drug-drug interactions. In the binary logistic regression model, the related factors identified in the bivariate analysis (p<0.05) were entered, and the odds ratio and 95% confidence interval were used to determine the independent risk factors for pDDIs. Statistical significance was p<0.05.

## Results

### Patient demographics

In total, 148 hospitalized patient case records were included during the study period, with 77 (52.02%) males and 71 (47.97%) females. Most patients were in the 21–40 age range (28.37%), followed by 61-80 years (27.70%). The mean age was 54.27±24.3 (Mean±SD), ranging from 18 to 107 years.

Among the patients, more than half (56.76%) had a medical history of one or more comorbidities. The most common were cardiovascular diseases (40.88%) followed by diabetes mellitus (28.72%) and dyslipidemia (7.18%). Respiratory tract infection (34.83%), urinary tract infection (34.19%), sepsis (14.8%), and gastroenteritis (7.09%) were the most common infectious diseases for hospital admission in our study. Most hospitalized patients had a stay duration of 6-10 days (56.08%), and the average length of stay was 8.16±2.85 days (range: 3-16 days). In our study, the majority of patients (45.27%) received 6–9 drugs per prescription, and the average number of drugs per prescription was 8.35±3.19 (Mean±SD) (range: 2-16) medications ( Table 1). ^35^

**Table 1.** Demographic details of the study population.

Variables	Categories	Frequency (%) (n=148)
Sex	Male	77 (52.02)
	Female	71( 47.97)
Age (in years)	≤20	10 (6.75)
	21–40	42 (28.37)
	41–60	30 (20.27)
	61–80	41 (27.70)
	81 and above	25 (16.89)
Number of Comorbidities	Nil	64 (43.24)
	1–2	49 (33.10)
	3–4	31 (20.94)
	Five or more	4 (2.70)
Types of Comorbidities	Diabetes mellitus	52 (28.72)
	Cardiovascular	74 (40.88)
	Dyslipidemia	13 (7.18)
	Renal diseases	06 (3.31)
	Neurologic	13 (7.18)
	Thyroid	02 (1.10)
	Hematologic	02 (1.10)
	Gastrointestinal	05 (2.76)
	Respiratory	02 (1.10)
	Others: Benign Prostate Hypertrophy (n=2) Chronic Liver Disease (n=3) Osteoarthritis (n=1) Tuberculosis (n=05)	12 (6.62)
Diagnosis	Respiratory tract infection	54 (34.83%)
	Urinary tract infection	53 (34.19%)
	Sepsis	23 (14.83%)
	Gastroenteritis	11 (7.09%)
	Others: Pancreatitis (n=3) Pyelonephritis (n=2) Pelvic Inflammatory Disease (n=2) Meningitis (n=1) Enteric Fever (n=1) Diarrhea (n=2) Food Poisoning (n=2) Ascites (n=1)	14 (9.03%)
Hospital Stay (days)	1–5	37 (25.0)
	6–10	83 (56.08)
	≥11	28 (18.91)
Drug Prescribed/Patient	2–5	28 (18.91)
	6–9	67 (45.27)
	≥10	53 (35.81)
Proportion of pDDIs	Total number of patients	148 (100%)
	Patients with at least one pDDI	99 (66.89%)

### Drug-drug interactions

In our study, 549 drug-drug interactions with 116 combinations of interacting drugs were observed. This includes 396 drug interactions from 64 non-antimicrobial combinations, 137 drug-drug interactions from 44 non-antimicrobial and antimicrobial combinations, and 16 drug-drug interactions from eight antimicrobial combinations. The mean drug interactions identified in the study population were 3.70±4.58 per prescription.

It was observed that 99 prescriptions were found to have the potential for at least one or more DDIs with a prevalence rate of 66.89%, irrespective of the type of severity. The identified DDIs classified according to severity show that most of the interactions, 220 (40.07%) belong to the ‘moderate’ category, 155 (28.23%) were minor/non-significant, and 145 (26.41%) were classified ‘minor.’ A total of 29(5.28%) drug interactions were rated as a ‘significant’ severity category ( Table 2).

**Table 2.** Types of drug combinations causing drug interactions identified in the study population.

Types of drug combinations with interacting pairs (n=116)	Level of Severity				Total number of DDIs (n=549) (%)	χ ^2^	P value
	Minor/non-significant (n=155) (%)	Minor (n=145) (%)	Moderate (n=220) (%)	Significant (n=29) (%)			
Non-antimicrobial agents *vs.* Antimicrobial agents (n=64)	115 (29.04)	95 (23.98)	167 (42.17)	19 (4.79)	396 (100)	37.52 ^†^	0.078
Non-antimicrobial agents *vs.* Non-antimicrobial agents (n=44)	38 (27.73)	47 (34.30)	44 (32.11)	08 (5.83)	137 (100)		
Antimicrobial agents *vs.* Antimicrobial agents (n=08)	02 (12.50)	03 (18.75)	09 (56.25)	02 (12.50)	16 (100)		

^†^ Fisher’s exact.

* p value <0.05 is statistically significant.

### Class of medications involved in causing drug interactions

**Figure 1.**
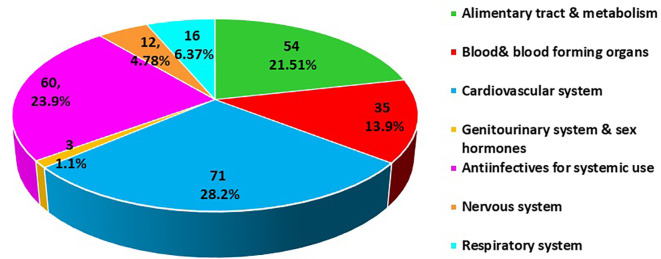
Anatomical Therapeutic Chemical (ATC) classification of drugs involved in potential drug-drug interactions (pDDIs).

The classification of pDDIs based on the Anatomical Therapeutic Classification (ATC) found a higher prevalence in the category cardiovascular system (28.8%) followed by anti-infective for systemic use (23.9%) and alimentary tract and metabolism (21.5%) ( Figure 1).

The evaluation of the underlying mechanism that causes DDIs showed that 210 (38.25) interactions involve pharmacokinetic interactions, while 181 (32.96%) interactions were caused by ‘others’ or unknown mechanisms. The remaining 158 (28.77%) interactions were known to be produced by pharmacodynamic interactions.

The most frequently identified DDIs were the combination of atorvastatin with clopidogrel, bisoprolol, amlodipine, or pantoprazole; aspirin with insulin; clopidogrel enoxaparin and furosemide with valsartan; and lisinopril and bisoprolol. The antimicrobial drugs involved in pDDIs were combinations of ceftriaxone with enoxaparin and aspirin, levofloxacin with insulin, furosemide, and piperacillin/tazobactam with aspirin, metformin, and doxycycline ( Table 3).

**Table 3.** Most common drug-drug interactions and their effect were identified among the study population.

Interaction	Drug combinations	Frequency	Effect	Level of concern	Source of recommendation
** Significant**	Aspirin – Enoxaparin	04	Both increase anticoagulation & Increase the risk of bleeding	04	pa
	Clopidogrel – Enoxaparin	08	Both increase anticoagulation & Increase the risk of bleeding	04	pa
	Bisoprolol – potassium chloride	04	Both increase serum potassium	04	a
	Valsartan – Sacubitril	04	Increased risk of renal impairment, hyperkalemia, hypotension,	04	a
	Metronidazole – levofloxacin	05	METRONIDAZOLE and LEVOFLOXACIN both increase QTc interval, Increased risk of long QT Syndrome, and possible Torsades de pointes	04	p
**Moderate**	Isoniazid – Rifampicin	05	Rifampin enhances the metabolism of isoniazid to hepatotoxic metabolites	03	a
	Levofloxacin – Furosemide	06	Increased risk of long QT Syndrome and possible Torsades de pointes	03	pa
	Bisoprolol – Aspirin	13	Both increase serum potassium	03	pa
	Amlodipine – Atorvastatin	16	Both levels probably increased – increased risk of arrhythmia, edema, myopathy, elevated liver function tests	03	pa
	Levodopa – piperacillin/tazobactam	04	Anticholinergics may enhance the therapeutic effects of LEVODOPA but may also exacerbate tardive dyskinesia. In high doses, anticholinergics may decrease the impact of LEVODOPA by delaying its GI absorption	03	a
**Minor**	Clopidogrel – Atorvastatin	14	Levels of Clopidogrel's active metabolite can be decreased	02	pa
	Insulin – levofloxacin	08	Insulin effects may be increased, and Quinolone antibiotic administration may result in hyper- or hypoglycemia	02	a
	Lisinopril – levodopa	03	LISINOPRIL effects may be increased Consider decreasing the dosage of an antihypertensive agent	02	a
	Aspirin – piperacillin/tazobactam	16	Both Piperacillin/Tazobactam (PIPERACILLIN) and ASPIRIN levels may be increased	02	a
	Clopidogrel – Amlodipine	05	Levels of CLOPIDOGREL's active metabolite can be decreased	02	pa
**Minor/non-significant **	Bisoprolol/atorvastatin	17	Bisoprolol levels can be slightly increased, and Increased risk of bradycardia	01	pa
	Furosemide/piperacillin & tazobactam	06	Both decrease cholinergic effects/transmission Increased risk of anticholinergic syndrome (dilated pupils, vasodilation/flushing, hyperthermia, dry skin)	01	p
	Metformin – furosemide	02	METFORMIN levels may be increased	01	a
	Memantine – metformin	01	Both drugs minimally increase the effects of the other drug involved in the mechanism	01	pa
	Clindamycin – piperacillin/tazobactam	02	CLINDAMYCIN and Piperacillin/Tazobactam (PIPERACILLIN) both decrease cholinergic effects/transmission Increased risk of anticholinergic syndrome (dilated pupils, vasodilation/flushing, hyperthermia, dry skin, hallucinations/agitation, constipation/urinary retention, tachycardia)	01	p

**Level of concern**: 01 - Minor or non-significant drug/drug interaction; 02 – Possible drug-drug interaction; 03 – Likely drug-drug interaction; 04 – Probable serious or life-threatening drug-drug interactions.

**Source of Recommendation (SOR)**: p - predicted drug/drug interaction based on pharmacokinetic or pharmacodynamic principles; a - drug/drug interaction in literature; pa - predicted and recognized drug/drug interaction based on pharmacokinetic or pharmacodynamic principles.

### Factors associated with pDDIs in the study population

Analysis of the factors related to the appearance of pDDIs showed that there was a statistically significant association with age, comorbidities, length of hospital stay, and the number of drugs prescribed (p<0.05) ( Table 4).

**Table 4.** Bivariate analysis of factors associated with potential drug-drug interactions among the study population.

Variables	Categories	Presence of DDIs [n(%)]	Absence of DDIs [n(%)]	χ ^2^	p-value
Sex	Male	56 (56.5)	21 (42.8)	2.46	0.116
	Female	43 (43.4)	28 (57.1)		
Age (in years)	≤20	5 (5.05)	5 (10.2)	13.82	**0.008** *
	21–40	20 (20.2)	22 (44.8)		
	41–60	21 (21.2)	9 (18.3)		
	61–80	33 (33.3)	8 (16.3)		
	81 and above	20 (20.2)	5 (34.6)		
Comorbidities	Present	69 (69.6)	15 (30.6)	20.40	**<0.001** **
	Absent	30 (30.3)	34 (69.3)		
Hospital stay (in days)	1–5	20 (20.2)	17 (34.6)	7.24	**0.027** *
	6–10	55 (55.5)	28 (57.1)		
	≥11	24 (24.2)	04 (8.1)		
Number of drugs prescribed	2–5	9 (9.09)	19 (38.07)	23.03	**<0.001** **
	6–9	45 (45.04)	22 (44.8)		
	≥10	45 (45.4)	8 (16.3)		

DDIs: drug-drug interactions.

* p<0.05 statistically significant.

** p<0.01 highly statistically significant.

In the binary logistic regression analysis, the dependent variable was the presence or absence of pDDIs, and the predictor variables were age, comorbidities, hospital stay, and the number of drugs prescribed. Drug-drug interactions were significantly associated with comorbidities and the number of medications prescribed (p<0.05) ( Table 5).

**Table 5.** Multiple binary logistic regression analysis for factors associated with potential drug-drug interactions among the study population.

Variables	P value	Adjusted OR	Odds ratio (95% CI)
Age (in years)	0.158	0.545	0.234–1.267
Comorbidities	**0.019** *	0.341	0.139–0.837
Hospital stay (in days)	0.338	1.721	0.567–5.223
Number of drugs prescribed	**0.025** *	0.244	0.071–0.838

* p<0.05 statistically significant.

## Discussion

Drug interactions contribute to undesirable health outcomes, compromise the clinical effectiveness of drug therapy, increase hospital visits, and prolong hospital stays. ^21^ The overall prevalence of pDDIs in our study was 67%, higher than the study by Hamdouk et al., who reported at least one pDDIs in 62.9%8% of the study sample. ^22^ Downward trends in prevalence were documented in earlier studies by Kuscu *et al*. (60%) and Rabba *et al*. (56%), respectively. ^23^^,^^24^

This disparity in the prevalence of pDDIs may be attributed to the differences in the study setting, study population, prescribing pattern of medications, and types of pDDIs and tools used to screen drug interactions in the study. In the present study, the average was 3.70±4.58 drug interactions per prescription among hospitalized patients. Documented evidence indicates that drug interactions occur more predominantly in hospitalized patients than in outpatients, considering the severity of the disease, comorbidities, and prescription of multiple medications with frequent modifications during their stay. ^25^

In the current study, aspirin, clopidogrel, statins, enoxaparin, furosemide, valsartan, and bisoprolol were prescribed to prevent and manage cardiovascular diseases. Documented evidence indicates that the use of these medications, either individually or in combination, is associated with various drug interactions, including increased bleeding, electrolyte imbalances, renal failure, and hypotension. ^23^^,^^25^^–^^27^ However, prescribing these medications is sometimes unavoidable and therapeutically valuable as a lifesaving medication. Therefore, close monitoring for effective treatment and evaluation of the benefit-risk assessment of actual DDIs of prescribed drugs is warranted. At the same time, careful laboratory assessment of international normalized ratio, serum electrolytes, renal and liver function tests, signs and symptoms of bleeding, and blood pressure monitoring are vital during treatment.

Similarly, metformin, sitagliptin, insulin, tamsulosin, memantine, levodopa, pantoprazole, paracetamol, and supplements such as potassium chloride and calcium carbonate were some of the important medications prescribed for the various other medical conditions in our study. In addition, drugs such as penicillins, cephalosporins, fluoroquinolones, metronidazole, macrolides, doxycycline, linezolid, isoniazid, rifampicin, vancomycin, and amphotericin B were some of the important antimicrobial agents used in this study. Drugs that cause enzyme induction or inhibition, resulting in reduced metabolism or clinical effects and alteration of gastrointestinal absorption, are the most common mechanisms related to antimicrobial interactions. ^28^

Cautious prescribing should be exercised when co-administering drugs with a narrow therapeutic index and drugs metabolized through cytochrome P450 isozymes that can develop clinically significant unpredictable drug interactions, particularly in patients with renal and hepatic impairment and the elderly population. ^29^^–^^31^ In the present study, the ATC class of medications involved in pDDIs showed a higher prevalence in the cardiovascular system (28.2%), followed by anti-infective for systemic use (23.9%). The increase in the prevalence of cardiovascular disease could be related to the use of complex medications for the long-term treatment of comorbidities and associated complications among the study populations. Our findings are consistent with those of Noor *et al*., Vazquez-Cornejo *et al.*, and Samardzic *et al*., who reported an increased prevalence of pDDIs in patients with cardiovascular disease. ^25^^,^^26^^,^^30^ Furthermore, an earlier study by Pavanello *et al.* in critical care patients showed that the most common drug class involved in pDDIs was anti-infective for systemic use, accounting for 45.8%, respectively. ^28^^,^^32^ The difference in study settings, varying profiles of study populations, disagreement in treatment guidelines and prescribing practice, and the use of different clinical decision support tools to analyze drug interactions may help explain the difference in the class of drugs involved in the onset of pDDIs.

In the present study, the severity level of most drug interactions was ‘moderate’ (40.0%) followed by ‘minor/non-significant’ (28.2%). Not all potential drug-drug interactions (pDDIs) are of equal severity, making the assessment of their severity crucial for recognizing their clinical significance and ensuring appropriate management. Only a small percentage (5.28%) of the identified drug interactions were found to be categorized as having a level of severity ‘significant,’ which requires close monitoring to avoid any adverse outcome of the pDDIs. It is suggested that a possible reason for the findings is that the risk factors and severity of potential drug-drug interactions (pDDIs) may be known to physicians, who might have tailored drug therapy to avoid or minimize these interactions.

These findings align with the results of the study by Noor *et al.* and Obeid *et al.*, who reported that most of the interactions were ‘moderate’ in severity. ^26^^,^^33^ Contrary to our findings, studies by Rabba *et al.* and Eneh *et al.* reported 66.4% and 52.7% of interactions with ‘major’ in severity level. ^24^^,^^29^ The difference in defining the classification and grading of severity between the resources could be a possible reason for the varying study results. Studies have observed that mechanism of action plays a significant role in DDIs, which requires management by either reducing the dose of one drug by 25% or 50%, changing the frequency of administration and dosage form, or avoiding such combination, replacing it with another medication. ^25^^,^^34^^,^^35^

Our study showed that ‘pharmacokinetic interactions’ were the most common underlying mechanism that caused pDDIs compared to pharmacodynamic and other unknown mechanisms. Similar observations have been cited in the study by Tesfaye *et al*., who reported pharmacokinetic interactions as the most common mechanism involved in causing pDDIs compared with pharmacodynamic and other/unknown interactions. ^36^

Studies have emphasized that patient characteristics such as age, comorbidities, number of medications prescribed, and hospital stay are risk factors for clinically significant pDDIs. ^35^^,^^37^^,^^38^ Age, comorbidities, length of hospital stay, and polypharmacy predispose patients to pDDIs. It is important to note that aging populations are at risk of developing multiple comorbid medical conditions that require frequent hospital visits and a prolonged stay prescribed with more complex therapeutic regimens. ^39^ Physiological changes related to age and variations in pharmacokinetics and pharmacodynamic parameters increase the risk and greater chance of developing pDDIs and adverse outcomes that reduce the efficacy of the treatment. ^40^ Pharmacists are essential in spotting possible drug-drug interactions (DDIs) and fixing them, which can greatly lower the chance of patient injury. Their participation in clinical decision-making and pharmaceutical therapy management is crucial for guaranteeing safe and efficient drug use, especially in intricate treatment plans.

### Strengths

The strength of our study includes the prospective observational design, which allowed for real-time data collection and assessment of potential drug-drug interactions (pDDIs) in a clinical setting. Additionally, a comprehensive electronic health record system enhanced data accuracy and completeness. The study’s focus on hospitalized patients with infectious diseases also provides valuable insights into medication management in this vulnerable population.

### Limitations

Our study has a few limitations. First, only one database would limit the number of pDDIs and may not reflect all pDDIs. Using multiple database tools and comparisons may help define the results more explicitly. Second, the data for the present study were collected from the Wareed system and mainly focused on the theoretical pDDIs. Due to a lack of follow-up, they could not address the drug interactions and results from a clinical viewpoint. Third, the study only included patients with specific indications in the internal general medicine ward. Therefore, the findings cannot be extended or applied to other specialty wards, intensive care units, or outpatient settings. Finally, this study is an observational design, which did not include an intervention. The lack of an intervention may have limited the ability to directly impact patient outcomes and reduce the incidence of drug-drug interactions (DDIs). Implementing a targeted intervention, such as clinical decision support or tailored drug therapy, could have potentially improved patient outcomes and minimized the occurrence of harmful DDIs.

## Conclusions

The present study identified a high frequency of pDDIs in hospitalized patients with infectious diseases. Antimicrobial agents and co-prescribed medications interacted; most of the interactions in our study had ‘moderate’ levels of severity. This study highlighted that advanced age, multiple comorbidities, and polypharmacy were independent risk factors for pDDIs. Knowledge about pDDIs and the regular use of professional drug information database support systems can help prescribers optimize drug therapy and enhance health outcomes. The study strongly recommends that regular review of patient drug therapy by a clinical pharmacist might avoid possible drug combinations that are likely to cause pDDIs and could ring a bell in improving the quality of patient-centered care.

## Data Availability

Figshare: ASSESSMENT OF POTENTIAL DRUG-DRUG INTERACTIONS IN HOSPITALIZED PATIENTS WITH INFECTIOUS DISEASES – AN EXPERIENCE FROM A SECONDARY CARE HOSPITAL. https://doi.org/10.6084/m9.figshare.24220714.v2. ^41^ Data are available under the terms of the Creative Commons Attribution 4.0 International license (CC-BY 4.0).
