# Supplementary material for: Assessment of potential drug-drug interactions in hospitalized patients with infectious diseases: an experience from a secondary care hospital
Source: F1000Res. 2025 Jan 2;13:164. Originally published 2024 Mar 8. [Version 4] doi: 10.12688/f1000research.143186.4 (PMC11809622; doi:10.12688/f1000research.143186.4)
Supplement: Peer review history

# Reviewer report: Reviewer response for version 4

**DOI:** 10.5256/f1000research.175825.r355297

**Published:** 2025-01-03

**Authors:** Abrar K Thabit

**Affiliations:** 1 Department of Pharmacy Practice, King Abdulaziz University, Jeddah, Saudi Arabia

I appreciate the authors' efforts in improving their manuscript. I approve the current version of the manuscript.

Is the work clearly and accurately presented and does it cite the current literature?

Partly

If applicable, is the statistical analysis and its interpretation appropriate?

Yes

Are all the source data underlying the results available to ensure full reproducibility?

No source data required

Is the study design appropriate and is the work technically sound?

Yes

Are the conclusions drawn adequately supported by the results?

Yes

Are sufficient details of methods and analysis provided to allow replication by others?

Partly

Reviewer Expertise:

Infectious diseases, clinical microbiology, clinical pharmacy, and academia.

I confirm that I have read this submission and believe that I have an appropriate level of expertise to confirm that it is of an acceptable scientific standard.

# Reviewer report: Reviewer response for version 3

**DOI:** 10.5256/f1000research.174389.r339909

**Published:** 2024-12-16

**Authors:** Émilie Bortolussi-Courval

**Affiliations:** 1 McGill University, Montreal, Québec, Canada

Thank you for the opportunity to review this manuscript again. Below are my comments. 

 Please provide references for pharmacodynamic and pharmacokinetic definitions. 

 In the Results section, would the authors prefer using two decimal points or one decimal point? In the mean age (sd), authors present a mean with 2 decimal points with an sd of one decimal point. Please pick either one or two decimal points and harmonize this throughout the manuscript. 

 Authors did not mention the need to use IQR in the methods (i.e. mean/sd or median/IQR were reported as required). There is no way to know if the authors presented mean/sd when the median/IQR would have been more appropriate. 

 Can authors please provide a distinction between pDDIs and DDIs? Both are used throughout the manuscript making it difficult to understand the difference in the text. 

 My main comment is that authors make no visible mention of the fact that covariates were selected a priori, although they state having done so in the Version 2 Amendments paragraph. Again, this is a crucial item to include in the manuscript. 

 Authors did not correct an important typo that I had highlighted in the text:

 What is "62.9%8%" ?

 In Table 3, under the Sources of recommendation, authors did not provide a legend to indicate what the different letters mean ("a", "pa", etc.)

 In Table 4, please harmonize one vs two decimal points. 

 What does the "number of drugs per prescription" mean? 

 Thank you for deleting Table 3. This new table is much clearer. 

 This text still requires substantial work.

Is the work clearly and accurately presented and does it cite the current literature?

No

If applicable, is the statistical analysis and its interpretation appropriate?

Partly

Are all the source data underlying the results available to ensure full reproducibility?

No

Is the study design appropriate and is the work technically sound?

Partly

Are the conclusions drawn adequately supported by the results?

No

Are sufficient details of methods and analysis provided to allow replication by others?

Yes

Reviewer Expertise:

Polypharmacy, deprescribing, medication safety, pharmacoepidemiology

I confirm that I have read this submission and believe that I have an appropriate level of expertise to state that I do not consider it to be of an acceptable scientific standard, for reasons outlined above.

# Response

**Published:** 2024-12-22

**Authors:** JAVEDH SHAREEF

**Affiliations:** CLINICAL PHARMACY AND PHARMACO, RAS AL KHAIMAH MEDICAL AND HEALTH SCIENCES UNIVERSITY, AL QUSAIDAT, RAS AL KHAIMAH, United Arab Emirates

**Please provide references for pharmacodynamic and pharmacokinetic definitions**

 We have now included citations for all the definitions provided in the Mechanism of Interaction section. (Ref. 19)

**In the Results section, would the authors prefer using two decimal points or one decimal point? In the mean age (sd), authors present a mean with 2 decimal points with an sd of one decimal point. Please pick either one or two decimal points and harmonize this throughout the manuscript.**

 Regarding the decimal points used in the Results section, I appreciate your insightful observation. We both believe that it's critical to convey numerical facts consistently. We have standardised the use of decimal points throughout the manuscript in response to your advice. To maintain uniformity, we have specifically decided to utilise [two] decimal points for all mean values and standard deviations (SDs). We think that this method improves the presentation of our findings in terms of uniformity and clarity. We value your attention to this detail and have made the required changes across the manuscript.

**Authors did not mention the need to use IQR in the methods (i.e. mean/sd or median/IQR were reported as required). There is no way to know if the authors presented mean/sd when the median/IQR would have been more appropriate**

 Acknowledge the reviewer’s concern that the methodology section did not mention when to use mean/SD or median/IQR.

 In our study, we used frequencies and percentages for categorical data and mean ± SD for continuous variables assuming a normal distribution. On the other hand, skewed or non-normally distributed data are better suited for the median and IQR. Since there was no discernible skewness in the data, we did not use them; however, you will examine the data once more to make sure that no variables should have been reported using median/IQR.

**Can authors please provide a distinction between pDDIs and DDIs? Both are used throughout the manuscript making it difficult to understand the difference in the text..**

 DDIs typically refer to confirmed or clinically observed drug-drug interactions whereas pDDIs refer to interactions that are identified based on theoretical or database-generated information, but have not yet been confirmed clinically in the study population. Thank you for pointing out the potential confusion between pDDIs and DDIs in the manuscript. Given that our study is observational and uses database tools to identify drug-drug interactions, the correct terminology in our context should be pDDIs (potential drug-drug interactions) rather than DDIs (drug-drug interactions).

**My main comment is that authors make no visible mention of the fact that covariates were selected a priori, although they state having done so in the Version 2 Amendments paragraph. Again, this is a crucial item to include in the manuscript..**

 Thank you for your insightful comment. We acknowledge that the manuscript does not clearly mention that covariates were selected a priori, which is an important methodological detail. This oversight will be addressed in the revised manuscript.

 To clarify, covariates for our analysis were selected a priori, based on the relevant literature, clinical relevance, and prior studies related to drug-drug interactions (DDIs/pDDIs). We intended to ensure that our analysis would be driven by established factors known to influence drug interactions, rather than performing post hoc selection. We agree that this detail is crucial to include in the manuscript for full transparency and methodological clarity.

**Authors did not correct an important typo that I had highlighted in the text:**

**What is "62.9%8%" ?**

 I appreciate you pointing up the manuscript's typo. The error where "62.9%8%" was mentioned has been found and fixed. "62.9%" is now the updated correct value. We value your attention to detail and really regret this oversight.

**In Table 3, under the Sources of recommendation, authors did not provide a legend to indicate what the different letters mean ("a", "pa", etc.)?**

 In response, we have added a footnote to **Table 3** to clarify the **Level of Concern** and **Source of Recommendation (SOR)** for each drug-drug interaction (DDI). The footnote includes the following information:

- **Level of Concern**:
  - 01 – Minor or non-significant drug/drug interaction
  - 02 – Possible drug-drug interaction
  - 03 – Likely drug-drug interaction
  - 04 – Probable serious or life-threatening drug/drug interaction
- **Source of Recommendation (SOR)**:
  - p – Predicted drug/drug interaction based on pharmacokinetic or pharmacodynamic principles
  - a – Drug/drug interaction in literature
  - pa – Predicted and recognized drug/drug interaction based on pharmacokinetic or pharmacodynamic principles

This footnote should help clarify how the DDIs are categorized and the source of their recommendation, addressing the point raised.

**In Table 4, please harmonize one vs two decimal points.?**

 Thank you for your helpful suggestion. We have reviewed and harmonized the decimal points in Table 4, ensuring consistency throughout the table by using [two] decimal points. We believe this improves the clarity and presentation of the data.

**What does the "number of drugs per prescription" mean?**

 The "number of drugs per prescription" refers to the total count of different medications prescribed in a single prescription to a patient during their hospital stay.

**Thank you for deleting Table 3. This new table is much clearer.**

 I appreciate your compliments. We are happy that the updated table is more useful and understandable. Since it has enabled us to present the data in a more succinct and comprehensible manner, we appreciate your idea to remove Table 3.

# Reviewer report: Reviewer response for version 3

**DOI:** 10.5256/f1000research.174389.r343772

**Published:** 2024-12-05

**Authors:** Abrar K Thabit

**Affiliations:** 1 Department of Pharmacy Practice, King Abdulaziz University, Jeddah, Saudi Arabia

This is a good study that evaluated potential DDIs among patients hospitalized with infectious diseases. It provides some valuable insights that could be overlooked by clinicians. However, several issues need to be addressed.

 Abstract:

 1.    In the methods, remove the text on ethical committee approval and statistical analysis. Instead, mention how you classified these DDIs based on severity since you commented on that in the conclusion.

 2.    Results: Also, briefly mention something about the severity level of the most common DDIs.

 Introduction:

 3.    Correct "Infectious infections" to Infectious diseases"

 Methods:

 4.    Assessment of drug-drug interactions: Please cite the reference based on which you classified the DDIs based on severity. Also, please remove this statement and move it to the data analysis section: "The collected data were scrutinized and checked for completeness, clarity, and legibility before being entered into a Microsoft Excel (RRID: SCR_016137) spreadsheet and were later analyzed using IBM SPSS Statistics (RRID: SCR_016479) version 27. (IBM Corp., Armonk, NY, USA)."

 5.    Severity level of the drug interaction: This section should be combined with the previous one as they're strongly related.

 6.    Mechanism of interaction: Again, cite the references of all the definitions.

 7.    Data analysis: mean and standard deviation are used to describe continuous data and not categorical data. Please correct.

 Results:

 8.    Table 1: Remove the "SI No" column.

 9.    Class of medications involved in causing drug interactions: The following statement should be moved to the methods with a citation of the website: "The analysis of the class of medications involved in the onset of pDDIs carried out using the Anatomical Therapeutic Chemical (ATC) classification system derived by the World Health Organization."

 10.    Figure 1: The figure appears blurry. Please improve the quality of the figure.

 11.    Table 5: Replace "Exp(B)" with "Adjusted OR". Also, the last column should be titled "95% confidence interval" only. I also suggest removing the following columns as they don't offer very useful information beside the adjusted OR, 95% CI, and P value: The beta coefficient of predictor variables, Standard error, and Wald. Also, remove the constant row.

 12.    In the methods, you mentioned pharmacokinetic and pharmacodynamic interactions. However, I don't see any classification of the reported DDIs based on these definitions. I suggest adding a column to Table 3 classifying each interaction into one of these two categories.

 Discussion:

 13.    This statement should be rephrased for clarity: "The current study prescribed aspirin, ..."

 14.    Rephrase this statement and make it in a passive tense: "The author suggests that ..."

 Limitations:

 15.    I think among the limitations is the lack of intervention given the observational study design. Making an intervention would have improved the patients outcomes and reduced the incidence of DDIs.

 Conclusion:

 16.    Replace "higher" with "high"

 17.    I suggest including a statement emphasizing the critical role of pharmacists on spotting these DDIs and potentially reducing patient harm by resolving them as possible.

 General comment:

 18.    I didn't see any interaction between fluoroquinolones and minerals, such as iron and calcium which are commonly given to hospitalized patients. I also wondered the lack of interactions between fluoroquinolones and macrolides with QT-prolonging agents, such as antiarrhythmics and antidepressants. Were these not reported at all?

Is the work clearly and accurately presented and does it cite the current literature?

Partly

If applicable, is the statistical analysis and its interpretation appropriate?

Yes

Are all the source data underlying the results available to ensure full reproducibility?

No source data required

Is the study design appropriate and is the work technically sound?

Yes

Are the conclusions drawn adequately supported by the results?

Yes

Are sufficient details of methods and analysis provided to allow replication by others?

Partly

Reviewer Expertise:

Infectious diseases, clinical microbiology, clinical pharmacy, and academia.

I confirm that I have read this submission and believe that I have an appropriate level of expertise to confirm that it is of an acceptable scientific standard, however I have significant reservations, as outlined above.

# Response

**Published:** 2024-12-09

**Authors:** JAVEDH SHAREEF

**Affiliations:** CLINICAL PHARMACY AND PHARMACO, RAS AL KHAIMAH MEDICAL AND HEALTH SCIENCES UNIVERSITY, AL QUSAIDAT, RAS AL KHAIMAH, United Arab Emirates

Abstract: 

 In the methods, remove the text on ethical committee approval and statistical analysis. Instead, mention how you classified these DDIs based on severity since you commented on that in the conclusion.

**Author Response: **We deleted the elements about the statistical analysis and ethical committee approval from the abstract. In addition, we updated the methods section to illustrate how the DDIs were categorized according to their severity level. In particular, we identified potential DDIs (pDDIs) using the PEPID database and then classified them based on severity levels.

 Results: Also, briefly mention something about the severity level of the most common DDIs.

**Author Response: **We have added a brief note about the severity levels of the most prevalent DDIs in the study to the Results section. To contextualize the clinical significance of these findings, we specifically emphasized the severity level distribution among the most commonly detected interactions.

 Introduction:

 Correct "Infectious infections" to Infectious diseases" 

**Author Response: **As suggested, we have changed the term "Infectious infections" to "Infectious diseases" in the Introduction section.

 Methods:

 Assessment of drug-drug interactions: Please cite the reference based on which you classified the DDIs based on severity. Also, please remove this statement and move it to the data analysis section: "The collected data were scrutinized and checked for completeness, clarity, and legibility before being entered into a Microsoft Excel (RRID: SCR_016137) spreadsheet and were later analyzed using IBM SPSS Statistics (RRID: SCR_016479) version 27. (IBM Corp., Armonk, NY, USA)."

**Author Response: **We have updated the Methods section with the relevant reference for the severity-based DDI classification.

 Following your recommendation, we have repositioned the statement about the data entry and analysis processes to the Data Analysis section for improved organization and clarity.

 Severity level of the drug interaction: This section should be combined with the previous one as they're strongly related.

**Author Response: **We have merged the two sections to enhance the organization and readability of the manuscript, as suggested.

 Mechanism of interaction: Again, cite the references of all the definitions.

**Author Response: **We have now included citations for all the definitions provided in the **Mechanism of Interaction** section.

 Data analysis: mean and standard deviation describe continuous data, not categorical data. Please correct

**Author Response: **We have updated the Data Analysis section to ensure that frequencies, percentages, or proportions are used appropriately when describing categorical data.

 Results:

  Table 1: Remove the "SI No" column. 

**Author Response: **As per your recommendation, we have removed the "SI No" column from Table 1. The table has been updated to reflect this change.

 Class of medications involved in causing drug interactions: The following statement should be moved to the methods with a citation of the website: "The analysis of the class of medications involved in the onset of pDDIs carried out using the Anatomical Therapeutic Chemical (ATC) classification system derived by the World Health Organization."

**Author Response: **We have relocated the statement about analyzing drug classes using the Anatomical Therapeutic Chemical (ATC) classification system to the Methods section and included references to the World Health Organization's (WHO) ATC classification system.

 Figure 1: The figure appears blurry. Please improve the quality of the figure

**Author Response: **We have improved the quality of the figure and replaced the original with a higher-resolution version to ensure it is clear and legible.

 Table 5: Replace "Exp(B)" with "Adjusted OR". Also, the last column should be titled "95% confidence interval" only. I also suggest removing the following columns as they don't offer useful information besides the adjusted OR, 95% CI, and P value: The beta coefficient of predictor variables, Standard error, and Wald. Also, remove the constant row.

**Author Response: **As you recommended, we have replaced "Exp(B)" with "Adjusted OR. " The last column has been renamed "95% Confidence Interval" to clarify the information presented. I have removed the columns for the beta coefficient of predictor variables, standard error, and Wald statistic, as these do not contribute additional useful information beyond the adjusted OR, 95% CI, and p-value. The "constant row" has also been removed from the table.

 In the methods, you mentioned pharmacokinetic and pharmacodynamic interactions. However, based on these definitions, I don't see any classification of the reported DDIs. I suggest adding a column to Table 3 to classify each interaction into one of these two categories.

**Author Response: **In response, we have added a footnote to **Table 3** to clarify the **Level of Concern** and **Source of Recommendation (SOR)** for each drug-drug interaction (DDI). The footnote includes the following information:

- **Level of Concern**:
  - 01 – Minor or non-significant drug/drug interaction
  - 02 – Possible drug-drug interaction
  - 03 – Likely drug-drug interaction
  - 04 – Probable serious or life-threatening drug/drug interaction
- **Source of Recommendation (SOR)**:
  - p – Predicted drug/drug interaction based on pharmacokinetic or pharmacodynamic principles
  - a – Drug/drug interaction in literature
  - pa – Predicted and recognized drug/drug interaction based on pharmacokinetic or pharmacodynamic principles

This footnote should help clarify how the DDIs are categorized and the source of their recommendation, addressing the point raised.

 This statement should be clarified: "The current study prescribed aspirin, ..."

**Author Response: **We have clarified the language by rephrasing it in response. The new phrase follows: "In the current study, aspirin, clopidogrel, statins, enoxaparin, furosemide, valsartan, and bisoprolol were prescribed for the prevention and management of cardiovascular diseases."

 Rephrase this statement and make it in a passive tense: "The author suggests that ..."

**Author Response: **We have rephrased the sentence in the passive voice as follows:

 "It is suggested that a possible reason for the findings is that physicians may be aware of the risk factors and severity of potential drug-drug interactions (pDDIs) and might have tailored drug therapy to avoid or minimize these interactions."

 Limitations:

 15.    I think among the limitations is the lack of intervention given the observational study design. Intervening would have improved the patient's outcomes and reduced the incidence of DDIs.

**Author Response: **I concur that the study's observational design restricts the application of an intervention that might enhance patient outcomes and lower the frequency of drug-drug interactions (DDIs). We have added the following to the drawbacks section per your suggestion: "This study's observational design, which lacked an intervention, is one of its drawbacks.

 Conclusion:

 Replace "higher" with "high"

**Author Response: **We have replaced "higher" with "high" in the **Conclusion** section, as recommended, to improve the clarity and accuracy of the statement

 I suggest including a statement emphasizing the critical role of pharmacists in spotting these DDIs and potentially reducing patient harm by resolving them as soon as possible.

**Author Response: **We have added the following statement to the **Discussion** section:

 "Pharmacists are essential in spotting possible drug-drug interactions (DDIs) and fixing them, which can greatly lower the chance of patient injury. Their participation in clinical decision-making and pharmaceutical therapy management is crucial for guaranteeing safe and efficient drug use, especially in intricate treatment plans.

 General comment:

 I didn't see any interaction between fluoroquinolones and minerals, such as iron and calcium, commonly given to hospitalized patients. I also wondered about the lack of interactions between fluoroquinolones and macrolides with QT-prolonging agents, such as antiarrhythmics and antidepressants. Were these not reported at all?

**Author Response: **In our study, we evaluated potential drug-drug interactions (pDDIs) using the PEPID database. The database may report different interactions and may not always consistently identify the same interactions. The severity levels of interactions (ranging from mild to severe) might also differ between databases, and some interactions found in one database might not be included in another.

 Even though patients in our study were prescribed macrolides, fluoroquinolones, and QT-prolonging medications such as antidepressants and antiarrhythmics, the PEPID database did not find any interactions between these medications. The criteria for evaluating possible interactions or the database's particular constraints could cause this. We acknowledge that the specific database employed may impact the identification of pDDIs, and this variability should be taken into account when interpreting the findings.

# Reviewer report: Reviewer response for version 2

**DOI:** 10.5256/f1000research.169291.r329857

**Published:** 2024-10-22

**Authors:** Émilie Bortolussi-Courval

**Affiliations:** 1 McGill University, Montreal, Québec, Canada

Thank you for the opportunity to review this manuscript. This prospective observational study analyzed potential drug-drug interactions (pDDIs) among hospitalized adults with an infectious disease. 

 Based on my assessment, I do not believe authors sufficiently cited literature. In the introduction, there should be citations for the definition of a DDI. When authors mention "studies have reported that age, polypharmacy (...)", they do not cite any study to support these sttements. In the methodology, I was expecting citations for the European Medicines Agency Guidelines. I was disappointed to see that there were no guidelines followed for reporting the findings of this study, such as the STROBE guidelines.

 Methods

 When, during the course of the study, were the covariates selected? Was selection done a priori or posthoc? This is a very important clarification to make. 

 Definitions would have been required for pharmacokinetic and pharmacodynamic interactions.

 Were median/IQR ever necessary to use in this study, in the case of demographic data that was not normally distributed?

 Women may have more pDDIs because of a baseline longer QT interval; would it be possible to stratify outcomes according to sex? 

 Results

 Were there significantly more patients in a particular age group versus another?

 I believe the prevalence of pDDIs should be described in the Results section of the manuscript. This is an important finding that the reader should appreciate quickly. 

 What does "the average number of drugs per prescription was 8.35±3.19 medications" mean? I'm not sure I understand.

 Table 3: I do not understand the utility of this table. It appears as though several non-antimicrobial drugs are listed alone. Would it be possible to only use Figure 1? I believe it is more telling of the classes of drugs that were involved rather than the list of agents involved. The other issues I have with Table 3 are that i) there is no indication of the frequency of involvement of each medication in an interaction, and ii) Amphotericin B should belong in the antimicrobial category, not the non-antimicrobial category. 

 In the discussion, "cautious prescribing should be exercised when co-administering drugs with a narrow therapeutic index (...)", I was expecting citations for this. Was this from UpToDate? If so, it would require a citation.

 "A possible reason could be that physicians are aware of the risk factors and severity of pDDIs" : has this been previously documented in the literature? If so, a citation would be needed here. 

 "It is important to note that aging populations are at risk of developing multiple comorbid medical conditions that require frequent hospital visits and a prolonged stay prescribed with more complex therapeutic regimens". Is there a citation for this?

 Would the authors be able to provide the proportion of patients with a pDDI in the Results section of Table 1?

 There are several typos within the discussion that need to be fixed.

 What does "62.9%8% of the study sample" mean?

 Please fix this sentence: "All pDDIs. are not identical in severity and evaluating their severity l is very important to recognize their clinical significance and proper management." 

 What are the strengths of this study? I was expecting to see this in the discussion. 

 I do not see the relevance of the following sentence with the rest of the paragraph: "A review of the published evidence suggests that fluoroquinolones and macrolides are associated with QT prolongation and may cause life-threatening arrhythmias." I would consider removing it. 

 Given the several oversights that I have seen within the manuscript (omission of citation of studies despite referencing of "several studies", adding an antimicrobial agent in a under "non antimicrobial agent" column by mistake, several typos in the discussion) that make the interpretation of the study findings difficult, I am not able to accept this manuscript at this time. This study has the potential to be an important addition to the literature in the field, but there are several issues with the methodology and results of the manuscript that require clarification.

Is the work clearly and accurately presented and does it cite the current literature?

No

If applicable, is the statistical analysis and its interpretation appropriate?

Partly

Are all the source data underlying the results available to ensure full reproducibility?

No

Is the study design appropriate and is the work technically sound?

Partly

Are the conclusions drawn adequately supported by the results?

No

Are sufficient details of methods and analysis provided to allow replication by others?

Yes

Reviewer Expertise:

Polypharmacy, deprescribing, medication safety, pharmacoepidemiology

I confirm that I have read this submission and believe that I have an appropriate level of expertise to state that I do not consider it to be of an acceptable scientific standard, for reasons outlined above.

# Response

**Published:** 2024-11-03

**Authors:** JAVEDH SHAREEF

**Affiliations:** CLINICAL PHARMACY AND PHARMACO, RAS AL KHAIMAH MEDICAL AND HEALTH SCIENCES UNIVERSITY, AL QUSAIDAT, RAS AL KHAIMAH, United Arab Emirates

**In the introduction, there should be citations for the definition of a DDI.**

 Thank you for your valuable feedback regarding the definition of drug-drug interactions (DDIs) in the introduction. In the updated manuscript, I have provided proper citations for the concept of a DDI, specifying the sources in the reference list ( **Ref. 01**) for clarity.

**When authors mention "studies have reported that age, polypharmacy (...)", they do not cite any study to support these statements.**

 I have reviewed the relevant literature and included primary studies as citations in the revised manuscript to substantiate these assertions (Ref. 6,7).

**In the methodology, I was expecting citations for the European Medicines Agency Guidelines.**

 I have added citations for the European Medicines Agency Guidelines in the revised manuscript to support our methodology accurately (Ref. 18).

**When, during the course of the study, were the covariates selected? Was selection done a priori or posthoc? This is a very important clarification to make.**

 Before data collection and analysis, the covariates were chosen based on current literature and the clinical significance of potential drug-drug interactions (pDDIs).

**Definitions would have been required for pharmacokinetic and pharmacodynamic interactions.**

 The updated manuscript has included precise definitions of pharmacokinetic and pharmacodynamic interactions.

**Were median/IQR ever necessary to use in this study, in the case of demographic data that was not normally distributed?**

 We appreciate your interest in presenting demographic data using the median and interquartile range (IQR).

 We appreciate your interest in presenting demographic data using the median and interquartile range (IQR). Normality tests indicated that age and length of hospital stay were roughly normally distributed, so we presented these variables' mean and standard deviation (SD).

 In future studies, we will consider using the median and IQR where data is skewed.

**Women may have more pDDIs because of a baseline longer QT interval; would it be possible to stratify outcomes according to sex?**

 While our current analysis did not include sex-specific stratification, we recognize that stratifying outcomes by sex could provide valuable insights. Future studies will consider this approach to understand pDDIs across demographic groups better.

**Were there significantly more patients in a particular age group versus another?**

 Our analysis found that most patients fell within the 21–40 and 61–80 age ranges, with significant associations (p<0.05) between these age categories and pDDIs. In future studies, we will clarify these findings and consider a further breakdown of age-related data to enhance our understanding of age as a factor in pDDIs.

**I believe the prevalence of pDDIs should be described in the Results section of the manuscript. This is an important finding that the reader should appreciate quickly.**

 We have modified the Results section to include the prevalence rate of pDDIs among our study population. Highlighting this important finding can enhance the clarity and impact of our manuscript.

**What does "the average number of drugs per prescription was 8.35±3.19 medications" mean? I'm not sure I understand.**

 The statement "the average number of drugs per prescription was 8.35±3.19 medications" means that, on average, each patient was prescribed about 8.35 medications, with ±3.19 indicating the standard deviation.

**Table 3: I do not understand the utility of this table. It appears as though several non-antimicrobial drugs are listed alone. Would it be possible to only use Figure 1? I believe it is more telling of the classes of drugs that were involved rather than the list of agents involved. The other issues I have with Table 3 are that i) there is no indication of the frequency of involvement of each medication in an interaction, and ii) Amphotericin B should belong in the antimicrobial category, not the non-antimicrobial category.**

 Thank you for your feedback regarding Table 3. Utility of Table 3: To enhance clarity, I have removed Table 3 and will rely solely on Figure 1 for a streamlined presentation of drug classes involved in pDDIs. Amphotericin B has been reclassified as an antimicrobial agent.

**In the discussion, "cautious prescribing should be exercised when co-administering drugs with a narrow therapeutic index (...)", I was expecting citations for this. Was this from UpToDate? If so, it would require a citation.**

 The revised manuscript has included citations to support this claim (Ref. 27-29). This information was obtained from the previous similar studies, I have properly cited it in the revised text.

**A possible reason could be that physicians are aware of the risk factors and severity of pDDIs": has this been previously documented in the literature? If so, a citation would be needed here.**

 This observation was an author’s assumption based on our study’s results rather than documented in previous studies. I have revised the text to clarify that this is an author’s hypothesis rather than a literature-supported statement.

 " **It is important to note that aging populations are at risk of developing multiple comorbid medical conditions that require frequent hospital visits and a prolonged stay prescribed with more complex therapeutic regimens". Is there a citation for this?**

 I have included relevant studies as citations supporting this claim in the revised manuscript ( **Ref. 37).**

**Would the authors be able to provide the proportion of patients with a pDDI in the Results section of Table 1?**

 Thank you for your suggestion. I calculated and included the proportion of patients with pDDIs in the revised manuscript in Table 1.

**Please fix this sentence: "All pDDIs. are not identical in severity and evaluating their severity l is very important to recognize their clinical significance and proper management."**

 Thank you for your feedback. The sentence has been revised in the manuscript for clarity.

**I do not see the relevance of the following sentence with the rest of the paragraph: "A review of the published evidence suggests that fluoroquinolones and macrolides are associated with QT prolongation and may cause life-threatening arrhythmias." I would consider removing it.**

 Thank you for your comment. The sentence about fluoroquinolones and macrolides has been removed to improve paragraph coherence.

**What are the strengths of this study? I was expecting to see this in the discussion.**

 I have added a detailed discussion of the study’s strengths in the revised manuscript to highlight our contributions to pDDI research.

# Reviewer report: Reviewer response for version 2

**DOI:** 10.5256/f1000research.169291.r312034

**Published:** 2024-08-29

**Authors:** Rina Das

**Affiliations:** 1 MM College of Pharmacy, Maharishi Markandeshwar University, Ambala, Haryana, India

The suggested changes and corrections are done in the revised version.

Is the work clearly and accurately presented and does it cite the current literature?

Yes

If applicable, is the statistical analysis and its interpretation appropriate?

I cannot comment. A qualified statistician is required.

Are all the source data underlying the results available to ensure full reproducibility?

No source data required

Is the study design appropriate and is the work technically sound?

Partly

Are the conclusions drawn adequately supported by the results?

Partly

Are sufficient details of methods and analysis provided to allow replication by others?

Partly

Reviewer Expertise:

infectious diseases, diabetes care, hypertension therapy, liver diseases, serum enzyme study

I confirm that I have read this submission and believe that I have an appropriate level of expertise to confirm that it is of an acceptable scientific standard.

# Reviewer report: Reviewer response for version 1

**DOI:** 10.5256/f1000research.156820.r289750

**Published:** 2024-06-28

**Authors:** Mahadev Rao

**Affiliations:** 1 Department of Pharmacy Practice, Manipal Academy of Higher Education (MAHE), Manipal, Karnataka, 576104, India

**Assessment of potential drug-drug interactions in hospitalized patients with infectious diseases: an experience from a secondary care hospital**

 The authors aimed to assess potential drug-drug interactions (pDDIs) among hospitalized patients with infectious diseases in a secondary care hospital.

1. There is an inconsistency in the mention of “pDDIs”, “DDI”, drug–drug interactions and “drug interaction” across the manuscript.
2. There is a significant flaw in the study objectives, design and results. The study objective states that “Therefore, the present study was carried out to assess pDDIs among hospitalized patients with infectious diseases receiving antimicrobial agents in a secondary care hospital.” However, the study also reports on the pDDIs between 2 non-antimicrobial agents. Many pDDIs reported in Table 4 are between 2 non-antimicrobial agents, which may not justify the title and objectives.
3. There is a fundamental flaw in this study. Table 1 reports that tuberculosis is present in 1 patient as a comorbidity among 148 hospitalized patients studied. Whereas Table 4 shows that the frequency of Isoniazid-Rifampicin pDDI is 5, which cannot be possible. Further, isoniazid is administered along with rifampicin, and other antitubercular agents such as pyrazinamide and ethambutol as a combined fixed dose formulation to TB patients.
4. To justify the title and the objectives, the authors may focus on the identification of the pDDIs between the antimicrobial agents and between antimicrobial agents and other drug categories. Identify the frequency of the top interacting antimicrobial agents and their mechanism and classify based on the infection categories.
5. Table 1 has listed on the different comorbidities in the 148 hospitalized patients. What are the comorbidities, where a higher frequency of pDDIs between antimicrobial agents and other drug categories were observed?
6. Since this is a scientific manuscript, the detailed elaboration on the need for obtaining necessary ethics committee approval from the hospital in a paragraph is not required as mentioned in the study setting.
7. Most of the statements mentioned in the patient demographics are a repetition of the Table 1. There is no need for repeating the results and table.
8. Also, many result statements are repeated in the discussion section.
9. The study aimed to assess the potential drug-drug interaction among hospitalized patients with infectious diseases using only a single electronic physician information database (PEPID) interaction tool (mentioned in limitation).
10. In the Table 5, for the column- presence and absence of DDI, n and % is mentioned in column title, but only n is present in the results. Also is it “Number of drugs prescribed” instead of “No drugs prescribed”?.
11. The current manuscript requires extensive editing of English language.

Is the work clearly and accurately presented and does it cite the current literature?

No

If applicable, is the statistical analysis and its interpretation appropriate?

I cannot comment. A qualified statistician is required.

Are all the source data underlying the results available to ensure full reproducibility?

No source data required

Is the study design appropriate and is the work technically sound?

No

Are the conclusions drawn adequately supported by the results?

No

Are sufficient details of methods and analysis provided to allow replication by others?

Partly

Reviewer Expertise:

Drug-drug interactions, clinical pharmacy, precision medicine

I confirm that I have read this submission and believe that I have an appropriate level of expertise to state that I do not consider it to be of an acceptable scientific standard, for reasons outlined above.

# Reviewer report: Reviewer response for version 1

**DOI:** 10.5256/f1000research.156820.r272895

**Published:** 2024-05-16

**Authors:** Rina Das

**Affiliations:** 1 MM College of Pharmacy, Maharishi Markandeshwar University, Ambala, Haryana, India

The authors have studied 148 patient case records to assess the potential drug-drug interactions in hospitalized patients with infectious diseases giving more emphasis on comorbidity cases.

 The manuscript addresses the potential drug-drug interactions in hospitalized patients with infectious diseases. Since there are many studies investigating this theme, it is not clear how this research adds to the current field.

 A statement of confirmation that the study conforms to recognised ethical standards should be included (for example: Declaration of Helsinki; US Federal Policy for the Protection of Human Subjects; European Medicines Agency Guidelines for Good Clinical Practice).

 The title of article states that ''Assessment of potential drug-drug interactions in hospitalized patients with **infectious diseases**''......but the result states majority of drug interaction with non-antimicrobial combinations, ''396 drug interactions 64 non-antimicrobial combinations''. When the work was focused on patients with infectious diseases, majority of cases should have been included DDIs with non-antimicrobial and antimicrobial combinations, or antimicrobial combinations.

 Is the study design appropriate and is the work technically sound?

 The method described is not detailed enough to warrant possibility of replication by the readers. Details of the experimental setting, timeline, research questions, and references of the methods/scales have to be better explained.

 Also no description of infectious diseases is provided whereas all comorbidities are elaborately described.

 The method of identification of drug-drug interaction results is not described.

 The text needs editing and language review, eg Polypharmacy is common among hospitalized patients with **infectious infections** .........

Is the work clearly and accurately presented and does it cite the current literature?

Yes

If applicable, is the statistical analysis and its interpretation appropriate?

I cannot comment. A qualified statistician is required.

Are all the source data underlying the results available to ensure full reproducibility?

No source data required

Is the study design appropriate and is the work technically sound?

Partly

Are the conclusions drawn adequately supported by the results?

Partly

Are sufficient details of methods and analysis provided to allow replication by others?

Partly

Reviewer Expertise:

infectious diseases, diabetes care, hypertension therapy, liver diseases, serum enzyme study

I confirm that I have read this submission and believe that I have an appropriate level of expertise to state that I do not consider it to be of an acceptable scientific standard, for reasons outlined above.

# Response

**Published:** 2024-07-16

**Authors:** JAVEDH SHAREEF

**Affiliations:** CLINICAL PHARMACY AND PHARMACO, RAS AL KHAIMAH MEDICAL AND HEALTH SCIENCES UNIVERSITY, AL QUSAIDAT, RAS AL KHAIMAH, United Arab Emirates

**COMPLIANCE TO REVIEWER COMMENTS**

**Manuscript title: **ASSESSMENT OF POTENTIAL DRUG-DRUG INTERACTIONS IN HOSPITALIZED PATIENTS WITH INFECTIOUS DISEASES – AN EXPERIENCE FROM A SECONDARY CARE HOSPITAL

**Authors: **JAVEDH SHAREEF, Sathvik Belagodu Sridhar1, Abu Nawa Ahmad Ismail1, Padma G.M. Rao1, Rashid Ain Ur2

**Reviewer – 1**

 Rina Das, MM College of Pharmacy, Maharishi Markandeshwar University, Ambala, Haryana, India

**Sl No**

**Reviewer (comments)**

**Corrections Made**

**Page No.**

**1. **The authors have studied 148 patient case records to assess the potential drug-drug interactions in hospitalized patients with infectious diseases giving more emphasis on comorbidity cases.

 The manuscript addresses the potential drug-drug interactions in hospitalized patients with infectious diseases. Since there are many studies investigating this theme, it is not clear how this research adds to the current field.

**Response:** Thank you for your valuable feedback. Our investigation of pDDIs among hospitalized patients with infectious illnesses in the UAE adds a novel perspective to the current literature. While there are several global studies on this issue, our research is relevant to the UAE healthcare system, considering local prescription patterns, common infectious illnesses, and distinct patient demographics. By evaluating 148 patient case records, focusing on comorbidities, we present insights specific to this geographical situation. This localized approach broadens our understanding of medication safety and management measures, particularly in the UAE, with practical implications for enhancing patient care and outcomes in our healthcare system.

 --

**2. **A statement of confirmation that the study conforms to recognised ethical standards should be included (for example: Declaration of Helsinki; US Federal Policy for the Protection of Human Subjects; European Medicines Agency Guidelines for Good Clinical Practice).

**Response: **Thank you for your feedback. We confirm that our study "Assessment of potential drug-drug interactions in hospitalized patients with infectious diseases: an experience from a secondary care hospital" adheres to recognized ethical standards. This study was performed per the principles outlined in the Declaration of Helsinki, the US Federal Policy for the Protection of Human Subjects (Common Rule), and the European Medicines Agency Guidelines for Good Clinical Practice.

 04

**3. **The title of article states that ''Assessment of potential drug-drug interactions in hospitalized patients with infectious diseases''......but the result states majority of drug interaction with non-antimicrobial combinations, ''396 drug interactions 64 non-antimicrobial combinations''. When the work was focused on patients with infectious diseases, majority of cases should have been included DDIs with non-antimicrobial and antimicrobial combinations, or antimicrobial combinations.

**Response:** Thank you for your insightful observation. Our study aimed to investigate pDDIs in hospitalized patients with infectious diseases and also looked at interactions with antimicrobial and non-antimicrobial medications. This divergence from the stated aims could be clarified by emphasizing the comprehensive nature of our investigation into pDDIs, encompassing interactions across all medication classes administered to the patient group. This approach aimed to thoroughly assess potential interactions that could impact clinical management in secondary care settings.

 The primary aim of our study was to investigate pDDIs involving antimicrobial drugs, which are important in treating infectious disorders. However, throughout the process, we discovered substantial interactions with non-antimicrobial drugs, which might have an equivalent impact on patient outcomes. Including these data gives a more comprehensive understanding of the drug management problems that hospitalized patients experience. Our work provides a stronger platform for improving clinical procedures and guaranteeing patient safety by recognizing and managing antimicrobial and non-antimicrobial pDDIs.

 --

**4. **The method described is not detailed enough to warrant possibility of replication by the readers. Details of the experimental setting, timeline, research questions, and references of the methods/scales have to be better explained.

 Also no description of infectious diseases is provided whereas all comorbidities are elaborately described.

 The method of identification of drug-drug interaction results is not described.

**Response:** The methodology section has been revised to include details about the study setting, ethical approval, inclusion/exclusion criteria, study duration, reference to the drug interaction tools used, and method of assessing drug-drug interactions.

 A description of infectious diseases is provided in the first paragraph of the introduction section.

 4-6 & 03

**5. **The text needs editing and language review, e.g., Polypharmacy is common among hospitalized patients with infectious infections .........

**Response: **All the grammatical and construction errors have been rectified with the help of an English language expert and incorporated in the updated version of the manuscript.

 ---

**6. **Is the work clearly and accurately presented and does it cite the current literature?

 Yes

 Thank you!

 --
